# Sex effects on the association between sarcopenia EWGSOP and osteoporosis in outpatient older adults: data from the SARCOS study

**DOI:** 10.20945/2359-3997000000087

**Published:** 2018-10-01

**Authors:** Alberto Frisoli, Fabíola Giannattasio Martin, Antonio Carlos de Camargo Carvalho, Jairo Borges, Angela T. Paes, Sheila Jean McNeill Ingham

**Affiliations:** 1 Universidade Federal de São Paulo Universidade Federal de São Paulo Seção de Cardiogeriatria São Paulo SP Brasil Seção de Cardiogeriatria, Disciplina de Cardiologia, Universidade Federal de São Paulo (Unifesp), São Paulo, SP, Brasil; 2 Universidade Federal de São Paulo Universidade Federal de São Paulo Escola Paulista de Medicina São Paulo SP Brasil Disciplina de Cardiologia, Escola Paulista de Medicina, Universidade Federal de São Paulo (EPM-Unifesp), São Paulo, SP, Brasil; 3 Universidade Federal de São Paulo Universidade Federal de São Paulo Departamento de Estatística São Paulo SP Brasil Departamento de Estatística, Universidade Federal de São Paulo (Unifesp), São Paulo, SP, Brasil; 4 Universidade Federal de São Paulo Universidade Federal de São Paulo Escola Paulista de Medicina São Paulo SP Brasil Medicina Física e Reabilitação, Escola Paulista de Medicina, Universidade Federal de São Paulo (EPM-Unifesp), São Paulo, SP, Brasil

**Keywords:** Bone aging, muscle, body composition

## Abstract

**Objective:**

The objective was to evaluate the association between sarcopenia (EWGSOP) and osteoporosis in older adults.

**Subjects and methods:**

This is a cross sectional analysis of a baseline evaluation of the SARCopenia and OSteoporosis in Older Adults with Cardiovascular Diseases Study (SARCOS). Three hundred and thirty-two subjects over 65 years of age were evaluated. Sarcopenia was determined by EWGSOP flowchart and Osteoporosis was established by WHO's criteria. Physical function, comorbidities and medications were evaluated.

**Results:**

Women were older (79.8 ± 7.2 years) than men (78.21 ± 6.7 years) (p = 0.042). Osteoporosis occurred in 24.8% of men, and in 42.7% of women (p < 0.001); sarcopenia occurred in 25.5% of men and in 17.7%, of women (p = 0.103). Osteoporosis was diagnosed in 68% of sarcopenic women, however only 20.7% (p = 0.009) of women with osteoporosis had sarcopenia; in older men, 44.7% of individuals with sarcopenia presented osteoporosis and 42.9% (p = 0.013) of men with osteoporosis showed sarcopenia. In an adjusted logistic regression analyses for sarcopenia, osteoporosis presented a statistically significant association with sarcopenia in men [OR: 2.930 (95% CI: 1.044-8.237; p = 0.041)] but not in women [OR: 2.081 (0.787-5.5; p = 0.142)]; in the adjusted logistic regression analyses for osteoporosis, a statistically significant association occurred in men [OR: 2.984 (95% CI: 1.144-7.809; p = 0.025)], but not in women [OR: 2.093 (0.962-3.714; p = 0.137)].

**Conclusion:**

According to sex, there are significant differences in the association between sarcopenia EWGSOP and osteoporosis in outpatient older adults. It is strong and significant in males; in females, despite showing a positive trend, it was not statistically significant.

## INTRODUCTION

Sarcopenia and osteoporosis are musculoskeletal clinical syndromes related to ageing, and are major public health concerns due to their likely bad outcomes. While the loss of bone mass increases the risk of fractures, loss of muscle mass and strength are strongly associated with a higher chance of falls (1,2). In turn, falls and fractures may lead to immobilization and a decrease in physical activity and these raise the odds of disability, hospitalization and the need for inpatient rehabilitation (3,4).

Studies have demonstrated the correlation between low muscle mass and muscle strength and low bone mineral density in postmenopausal women (5) but not in men (6). However, the effect of bone on muscle mass and muscle strength has also been documented. Juffer and cols. (7) has shown that osteocytes stimulated by mechanostatic action produce a number of factors, such as IGF-I, MGF, VEGF, and HGF which stimulate muscular function and formation. More recently, Yoshimura and cols. (8) showed that osteoporosis predicts incidental sarcopenia by the Asian Work Group for Sarcopenia (AWGS) in 4 years, but contrary to expectations, the opposite relationship was not significant. These results presented great variation due to the diverse concepts of sarcopenia, as well as due to uncontrolled variables that do not permit the establishment of a clear association between sarcopenia and osteoporosis with old age and gender. Based on these findings, we hypothesized that sarcopenia, by EWGSOP (European Working Group on Sarcopenia in Older People), has a strong association with osteoporosis in older females but not in older males; additionally, we believe that the inverse association, i.e. osteoporosis with sarcopenia by EWGSOP will show the same trend in both sexes. To test our hypothesis, we evaluated the association between osteoporosis according to the WHO's definition (9) and sarcopenia according to the EWGSOP (10) in older men and women from the same population group, with established risk factors and significant variables that could interfere with bone mineral density, muscle mass and muscle strength.

## SUBJECTS AND METHODS

### Subjects

This study is a cross sectional analysis of a baseline evaluation of the SARCopenia and OSteoporosis in Older Adults with Cardiovascular Diseases Study (SARCOS), a one-year prospective cohort study that investigated the association between cardiovascular diseases and changes in body composition, muscle strength and physical performance as a common pathway to disability. We interviewed 383 older outpatient adults from an outpatient cardio-geriatric clinic and 332 were included in this study and underwent DXA analyzes.

Our population was composed by older adults, over 65 years of age, both sexes and all ethnic groups. Exclusion criteria were: unstable medical conditions, any form of cancer in the last five years, chronic renal failure in dialysis, Parkinson's disease, severe infectious disease requiring hospitalization in the previous month, moderate or severe dementia classified by the MMSE (mini-mental state examination) (11,12) and use of gait assistant devices. This study was approved by the Ethical Review Board at our Institution and written informed consent was obtained from all participants.

### Diagnosis of sarcopenia

Sarcopenia, as determined by EWGSOP's flowchart (10), is defined by the presence of weakness represented by grip strength of the dominant hand lower than 20 kgf for women and 30 kgf for men and/or a gait speed lower than 0.8 m/s, plus low appendicular muscle mass by height^2^ lower than 5.45 kg/m^2^ for women and 7.26 kg/m^2^ for men.

### Handgrip strength

Isometric grip strength of the dominant upper extremity was determined by three measurements with a handheld dynamometer (Jamar; TEC, Clifton, NJ, USA); maximum values are reported.

### Bone mineral density and osteoporosis

Bone mineral density (BMD – g/cm^2^) of the lumbar spine, femoral neck, total femur and appendicular muscle mass and total fat mass were assessed through a DXA analysis by dual-energy X-ray absorptiometry (GE Lunar; DPX-MD 73477, GE Medical system, Madison, WI). Osteoporosis was established by the WHO's criteria (9), *i.e.*, BMD T score ≤ −2.5 standard deviations (SD) at lumbar spine, femur neck, and total femur.

### Disability

Disability was assessed by the number of tasks performed in activities of daily living (ADL) and instrumental activities of daily living (IADL); the cut point for disability was 5 for ADL or 25 for IADL (13-15).

### Other measurements

Demographic data, weight, height, cardiovascular disease (arterial hypertension, atrial fibrillation, previous myocardial infarction, chronic atherosclerosis coronary, heart failure, peripheral arterial obstruction) and other chronic diseases: diabetes mellitus osteoarthritis, non-dialysis dependent chronic kidney disease (CKD), chronic obstructive pulmonary disease (COPD), previous diagnosis of cancer, current or previous consumption of alcohol (at least one year without drinking alcohol), and current or previous smoking history (at least five years without smoking). We also considered falls (at least one fall in last 6 months) and history of fractures (a clinical fracture or diagnosed by radiograph assessment in a Health Care service). Finally, we evaluated all medications that could interfere in bone and muscle metabolism, such as bisphosphonates, tereparatide, strontium ranelate, corticosteroids in high doses (≥ 7.5 mg predinisone/day or equivalent for more than 3 months), vitamin D over 800 IU/day, estrogen and progesterone replacements, ACE (angiotensin-converting-enzyme) inhibitors, ARB (angiotensin II receptor blocker) I and II inhibitors.

### Statistical analysis

Qualitative variables are expressed as absolute and relative frequencies. Quantitative data are summarized as means, medians, standard deviations, minimum and maximum values. To compare the groups (sarcopenia and osteoporosis) the *chi-square* test for qualitative variables and ANOVA for quantitative variables were used to compare differences in baseline characteristics by gender.

Binomial logistic regression analyses were performed to evaluate the association between osteoporosis (OP) with sarcopenia EWGSOP and vice versa. Adjusted regression analyses were performed with significant variables for sarcopenia (current use of ACE inhibitor/ARB I and II inhibitors, falls, previous fracture (only for women), diabetes mellitus and disability), and for osteoporosis (falls, age, previous alcohol consumption, disability, smoking history, bisphosphonate use and diabetes mellitus). In the case of two quantitative variables, scatter diagrams and correlation coefficients were used. SPSS version 22 (SPSS, Inc., Chicago, IL, USA) statistical software package was used for carrying out all the analyses. Statistical significance was set at 0.05.

## RESULTS

Demographic data, body composition parameters, muscle strength, prevalence of chronic and cardiovascular diseases, osteoporosis, and sarcopenia are described in Table 1.

**Table 1:** Demographic data, body composition parameters, muscle strength, prevalence of chronic and cardiovascular diseases, osteoporosis, and sarcopenia EWGSOP

	All (N = 332)	Men (N = 141)	Women (N = 191)	P value
Age (years) (average (SD))	78.44 (7.16)	78.21 (6.78)	79.81 (7.24)	0.042
Years of education (average (SD))	3.52 (3.03)	4.54 (3.51)	3.48 (2.94)	0.003
Personal income (average (SD))	1.63 (1.79)	2.13 (2.50)	1.24 (0.90)	< 0.001
Number of medications (average (SD))	6.48 (2.61)	6.20 (2.29)	6.65 (2.80)	0.124
Grip strength (kgf) (average (SD))	22.56 (7.86)	28.58 (6.49)	17.63 (4.78)	< 0.001
Lumbar spine BMD (g/cm^2^) (average (SD))	1.075 (0.223)	1.182 (0.22)	0.993 (0.20)	< 0.001
Femur neck BMD (g/cm^2^) (average (SD))	0.830 (0.154)	0.883 (0.16)	0.781 (0.13)	< 0.001
Total femur BMD (g/cm^2^) (average (SD))	0.871 (0.168)	0.931(0.16)	0.806 (0.14)	< 0.001
Total body fat (%) (average (SD))	39.62 (9.62)	33.88 (7.75)	44.16 (8.48)	< 0.001
IAMM (kg/m^2^) (average (SD))	6.74 (3.33)	7.21 (0.84)	6.00 (0.86)	< 0.001
Prior diagnosis of chronic diseases				
Hypertension (%)	92.1	92.2	92.0	0.832
Diabetes mellitus (%)	39.8	39.5	42.2	0.713
Previous consumption of alcohol (%)	16.3	33.9	2.6	< 0.001
Myocardial infarction (%)	32.6	42.7	26.9	0.007
Heart failure (%)	28.9	28.2	30.3	0.791
Previous diagnosis of osteoporosis (%)	20.9	6.5	31.4	< 0.001
COPD (%)	8.4	10.7	6.7	0.194
Previous fracture (%)	31.4	33.1	30.1	0.602
CKD (%)	16.3	16.9	14.8	0.743
Osteoarthritis (%)	37.3	22.0	46.8	< 0.001
Smoking history (%)	49.7	69.4	32.1	< 0.001
Disability	44.8	57.1	42.9	0.548
Falls in last 6 months (%)	27.1	16.3	30.8	0.007
Stroke (%)	15.4	15.3	16.0	1.061
Present diagnosis of sarcopenia and osteoporosis				
Sarcopenia EWGSOP (%)	18.4	25.5 (36)	17.7 (28)	0.103
Osteoporosis (OP) (%)	36	24.8 (35)	42.7 (82)	< 0.001

Personal income: US$ 312.50/month; IAMM: index of appendicular muscle mass (AMM/height^2^). Sarcopenia by EWGSOP (European Working Group on Sarcopenia in Older People); OP: osteoporosis at proximal femur and/or lumbar spine. COPD: chronic obstructive pulmonary disease; CKD: chronic kidney disease. P values refer to the difference between men and women.

### Prevalence of osteoporosis and sarcopenia

The characteristics of the older adults with and without osteoporosis and sarcopenia are shown in Tables 2 and 3.

**Table 2:** Demographic data and prevalence of chronic and cardiovascular diseases, in men and women with and without osteoporosis

	Men			Women		
	Non-Osteoporosis	Osteoporosis	p	Non-Osteoporosis	Osteoporosis	p
Subject characteristics						
Age (years) (average (SD))	77.3 (6.4)	81.8 (7.3)	< 0.001	78.7 (6.6)	80.9 (7.8)	0.049
Caucasians (%)	60.4	71.9	0.405	72.6	71.2	0.377
Smoking history (%)	67.2	65.4	1.054	23.4	33.9	0.282
Previous consumption of alcohol (%)	31.1	42.3	0.331	0	5.4	0.241
Falls in last 6 months (%)	16.4	19.2	0.764	27.7	26.8	1.003
Lumbar spine BMD (g/cm^2^) (average (SD))	1.230 (0.20)	0.992 (0.15)	< 0.001	1.119 (0.15)	0.844 (0.12)	< 0.001
Femur neck BMD (g/cm^2^) (average (SD))	0.935 (0.14)	0.735 (0.10)	< 0.001	0.870 (0.10)	0.683 (0.10)	< 0.001
Total femur BMD (g/cm^2^) (average (SD))	0.990 (0.14)	0.781 (0.12)	< 0.001	0.906 (0.11)	0.705 (0.11)	< 0.001
IAMM (kg/m^2^) (average (SD))	7.376 (0.88)	6.704 (0.93)	< 0.001	6.263 (0.94)	6.571 (6.52)	0.627
Total body fat (%) (average (SD))	34.78 (7.22)	31.00 (8.95)	0.009	45.36 (7.61)	42.14 (9.18)	0.008
Dominant grip strength (kgf) (average (SD))	29.4 (6.5)	26.2 (6.6)	0.022	18.5 (4.7)	16.7 (5.0)	0.027
Comorbidities						
Hypertension (%)	92.3	84.4	0.296	91.6	90.4	1.000
Diabetes mellitus (%)	47.3	18.8	0.006	48.2	34.7	0.104
Heart failure (%)	25.3	37.5	0.254	66.3	26.0	0.382
Osteoarthritis (%)	23.3	18.8	0.632	47.6	45.2	0.873
Previous stroke (%)	13.2	18.8	0.561	14.3	17.8	0.663
Chronic kidney disease (%)	16.5	18.8	0.788	15.7	13.7	0.823
Previous cancer (%)	14.3	28.1	0.107	14.3	6.80	0.198
Disability (%)	37.2	48.7	0.256	38.4	60.5	0.003
History of osteoporosis (%)	3.3	15.6	0.028	27.4	35.6	0.302
Previous fractures (%)	27.5	50.0	0.029	27.4	32.9	0.488

Note: P values refer to the difference between men and women with and without osteoporosis.

IAMM: index of appendicular muscle mass (AMM/height^2^).

**Table 3:** Demographic data, body composition parameters, muscle strength, prevalence of chronic and cardiovascular diseases, of men and women with and without sarcopenia EWGSOP

	Men			Women		
	Non-Sarcopenia	Sarcopenia	p	Non-Sarcopenia	Sarcopenia	p
Subject characteristics						
Age (years old) (average (SD))	76.90 (6.20)	82.91 (7.00)	< 0.001	79.02 (6.95)	82.64 (8.06)	0.016
Caucasians (%)	60.4	71.9	0.059	70.8	78.6	0.331
Smoking history (%)	70.3	69.4	1.002	33.5	36.0	0.822
Previous consumption of alcohol (%)	31.4	33.3	0.865	3.4	4.0	1.009
Falls in last 6 months (%)	17.8	34.3	0.050	27.9	48.0	0.062
Lumbar spine BMD (g/cm^2^) (average (SD))	1.185 (0.22)	1.139 (0.21)	0.238	1.014 (0.19)	0.926 (0.23)	0.043
Femur neck BMD (g/cm^2^) (average (SD))	0.915 (0.15)	0.822 (0.16)	0.003	0.804 (0.13)	0.686 (0.10)	< 0.001
Total Femur BMD (g/cm^2^) (average (SD))	0.971 (0.16)	0.873 (0.17)	0.002	0.839 (0.14)	0.709 (0.12)	< 0.001
IAMM (kg/m^2^) (average (SD))	7.57 (0.80)	6.24 (0.64)	< 0.001	6.62 (4.63)	4.97 (0.36)	0.076
Total body fat (%) (average (SD))	33.82 (7.46)	33.48 (8.67)	0.825	44.37 (8.34)	40.97 (9.36)	0.063
Dominant grip strength (kgf) (average (SD))	31.08 (5.27)	21.41 (4.76)	< 0.001	18.67 (4.58)	12.92 (3.28)	< 0.001
Comorbidities						
Hypertension (%)	89	93.8	0.512	91.1	86.1	0.512
Diabetes mellitus (%)	45.1	25.0	0.431	45.6	22.2	0.022
Heart Failure (%)	29.7	25.0	0.654	27.8	33.3	0.660
Osteoarthritis (%)	22.2	21.9	1.010	23.1	16.7	0.473
Previous stroke (%)	13.2	18.8	0.562	12.7	22.2	0.267
Chronic kidney disease (%)	15.4	21.9	0.422	15.2	16.7	1.000
Previous cancer (%)	14.3	28.1	0.101	13.9	30.6	0.043
Disability (%)	31.4	77.8	< 0.001	40.8	84.0	< 0.001
History of osteoporosis (%)	5.5	9.4	0.687	31.5	28.6	0.821
Previous fractures (%)	30.8	40.6	0.383	25.3	47.2	0.031

Note: P values refer to the difference between men and women with and without osteoporosis.

IAMM: index of appendicular muscle mass (AMM/height^2^).

### The association of osteoporosis and sarcopenia EWGSOP in older men and women

Osteoporosis was diagnosed in 52.5% (n = 32; p = 0.002) of subjects with sarcopenia, but sarcopenia was only diagnosed in 27.4% of subjects with osteoporosis. This trend was observed in women, where 68% of sarcopenic patients showed osteoporosis, and only 20.7% (n = 17; p = 0.009) of osteoporotic patients showed sarcopenia; contrary to this, 44.7% of men with sarcopenia presented with osteoporosis and 42.9%; (n = 15; p = 0.013) of men with osteoporosis presented with sarcopenia. Both disorders occurred in 10.6% of men and 8.9% of women.

In the logistic regression analyses for sarcopenia, osteoporosis presented a similar value of the association for men OR=3.03 (95% CI: 1.334-6.909; p = 0.008) and women OR = 3.30 (95%CI: 1.347-8.091; p = 0.009), and vice versa. In the adjusted logistic regression analyses, for sarcopenia and osteoporosis, we used different variables for, for men and women, according to statistical significance that they had presented previously (Tables 2 and 3). Variables used in the logistic regression analyses for sarcopenia in the female group were: age, previous clinical fractures, diabetes mellitus, falls in the last 6 months, cancer history, ACE/ARB I and II use and disability; for the male group, the same variables were used, with the exception of previous clinical fracture and cancer history. In the osteoporosis analyzes, the variables used in the female group were: age, smoking history, diabetes mellitus, falls in the last 6 months, previous consumption of alcohol, current use of bisphosphonates and disability; for the male group the same variables were used, with the exception of disability.

After the adjustment, osteoporosis presented a significant association with sarcopenia only in men with an OR: 2.930, (95% CI: 1.04-8.23; p = 0.041) and this trend remained in the analyses for osteoporosis, where sarcopenia presented an OR: 2.984 (1.144-7.809; p = 0.025). While in women, despite the analysis showing a positive trend in the association between osteoporosis and sarcopenia (OR: 2.081 (0.787-5.5; p = 0.142)), and vice versa (OR: 2.093 (0.962-3.714; p = 0.137)) they did not reach statistical significance (Table 4). Interestingly, contrary to the previous literature (10,16,17), age was not an independent predictor of osteoporosis and sarcopenia, in both sexes. Diabetes mellitus was negatively associated with osteoporosis in men, but in women this association was found to be inverse and also significant (Table 4). Disability showed the highest association with sarcopenia in both genders in comparison with the other variables. Finally, in women, previous fractures were also associated with sarcopenia (Table 4).

**Table 4:** Adjusted logistic regression analyses for sarcopenia EWGSOP in older women and men

	Sarcopenia EWGSOP in Men OR (95% CI; p)	Sarcopenia EWGSOP in Women OR (95% CI; p)
Osteoporosis	2.930 (1.044-8.237; **p = 0.041**)	2.081 (0.787-5.5; p = 0.142)
Age	1.053 (0.98-1.132; p = 0.161)	1.051 (0.982-1.125; p = 0.151)
Previous fractures		0.894 (0.328-2.441; p = 0.828)
Diabetes mellitus	2.462 (0.926-6.549; p = 0.071)	0.971 (0.36-2.621; p = 0.954)
Falls in last 6 months	1.635 (0.585-4.567; p = 0.349)	2.164 (0.85-5.511; p = 0.105)
Cancer history		0.467 (0.054-4; p = 0.487)
ACE inhibitor/ARB I and II inhibitors current use	1.333 (0.516-3.442; p = 0.553)	0.597 (0.211-1.684; p = 0.329)
Disability	6.546 (2.476-17.273; **p < 0.001**)	4.904 (1.487-16.172; **p = 0.009**)

Note: Adjusted logistic regression – variables used in the female group were: age, previous clinical fractures, diabetes mellitus, falls in the last 6 months, cancer history, ACE/ARB I and II use and disability; for the male group, the same variables were used, with the exception of previous clinical fracture and cancer history.

## DISCUSSION

To the best of our knowledge, this is the first study to demonstrate that there are important differences in the association between sarcopenia EWGSOP and osteoporosis in older adults determined by sex. Contrary to our hypothesis, females did have an association between osteoporosis and sarcopenia EWGSOP; however, this association did not remain relevant after adjustments for confounder clinical variables were performed.

Previous studies (18,19) on the relationship between loss of muscle mass and strength and osteoporosis have shown great variation according to sex, age and health, diagnostic criteria for sarcopenia and cutoff points used for bone loss; these factors may cause a significant variation in the association values. One of the few studies that used the EWGSOP's criteria analyzed 409 independent women, aged 70-80 years from the community, and did not find a significant association between low BMD and sarcopenia (20) although the prevalence of sarcopenia by EWGSOP was very low (0.9%) and only 36% of those women presented with osteopenia.

Our findings in women differ significantly from others, but the population, the definition of sarcopenia, and confounder variables evaluated were different. In the Osteoporosis Risk Factor and Prevention (OSTPRE) Study, women with sarcopenia by EWGSOP had 12.9 times (3.1–53.5; p < 0.001) higher odds of having osteoporosis when compared to women without sarcopenia; but in the OSTPRE study the sample was composed by younger (68.7 ± 1.8 yo) postmenopausal women from the dwelling community. Besides, in our outpatient population, sarcopenia diagnosis was made with a higher cut off for lean mass (cut-off of 6.3 kg/m^2^) (21). Another key point of our data was the diversity in the correlation between diabetes and osteoporosis among the sexes. In older women, the presence of diabetes has shown a higher risk of osteoporosis, whereas in men it appears to have a protective effect. However, these results should be evaluated with caution, since the study was not designed for this purpose. Higher levels of BMD in men with diabetes compared to non–diabetes subjects were also described in the Rotterdam, EVOS and The Health ABC studies; they have demonstrated 3-5% higher bone site BMD in men with diabetes vs. non-diabetes (22-24).

The greater tendency of osteoporosis in women, evidenced in our series and also present in other studies, may be justified by the earlier estrogenic deprivation caused by menopause, by the other hormone deficiencies, more comorbidities (25,26), and by the process of inflammaging (27). In men, osteoporosis usually begins during the seventh decade (28) justifying the difference in prevalence between sexes due to the decrease of testosterone. Estrogen deprivation, also affects the incidence of sarcopenia, but mainly by the loss of muscle strength (29). This theory is endorsed by studies noting that muscle strength is preserved in women who opt for hormone replacement therapy at the onset of menopause, as compared with those who do not (30).

We believe that screening for osteoporosis in older adult outpatients should be recommended not only to evaluate the risk of fractures through the analysis of BMD but additionally, to evaluate the risk of sarcopenia, an important risk factor for falls and fractures. This strong association between osteoporosis and sarcopenia, and vice versa, especially in older men, must be considered when deciding upon therapeutic strategies for the prevention of fractures, since it is of utmost importance that both conditions are treated.

This study has limitations. First, our sample size is small, although it is considerable if we concede that it is a very old population (average age 80 yo). Another limitation of our study was the non-radiological confirmation of bone fractures of the majority of patients who reported a history of fracture, which may cause a bias on the analyses of the osteoporotic sample, since previous fractures should be considered as having osteoporosis, independent of DXA. Also, as the average age is high, we cannot extrapolate our findings to a younger population. This study is a cross sectional analyses and, as such, does not allow us to establish a cause and effect relationship between the loss of BMD and the loss of appendicular muscle mass and/or muscle strength. We did not quantify myokines that could help shed some light on the interaction between loss of bone and muscle mass/strength.

In summary, older adults from an outpatient clinic setting presented with a high prevalence of osteoporosis and/or sarcopenia EWGSOP. In regards to sex, sarcopenia EWGSOP was more prevalent in males while osteoporosis was more prevalent in females. The interaction between muscle mass and muscle function with bone metabolism seems to be more intense in older men than in older women. In conclusion, according to sex, there are significant differences in the association between sarcopenia EWGSOP and osteoporosis in outpatient older adults. It is strong and significant in males; in females, despite showing a positive trend, it was not statistically significant.
